# Nursing Students’ Experiences of Clinical Practices in Emergency and Intensive Care Units

**DOI:** 10.3390/ijerph17165686

**Published:** 2020-08-06

**Authors:** María González-García, Alberto Lana, Paula Zurrón-Madera, Yolanda Valcárcel-Álvarez, Ana Fernández-Feito

**Affiliations:** 1 Health Care Service of Asturias, Central University Hospital of Asturias, Avda. Roma, s/n, 33011 Oviedo, Spain; maguig87@hotmail.com (M.G.-G.); yolanda.valcarcel@sespa.es (Y.V.-Á.); 2 Department of Medicine, School of Medicine and Health Sciences, University of Oviedo, Avda. Julián Clavería, s/n, 33006 Oviedo, Spain; zurronpaula@uniovi.es (P.Z.-M.); fernandezfana@uniovi.es (A.F.-F.); 3 Healthcare Research Area, Health Research Institute of Asturias (ISPA), Avda. Roma, s/n, 33011 Oviedo, Spain; 4 Mental Health Center of La Corredoria, Health Care Service of Asturias (Spain), C. Alfredo Blanco, s/n, 33011 Oviedo, Spain

**Keywords:** clinical placements, emergency hospital service, intensive care units, nursing care, nursing education research, nursing students, nursing

## Abstract

Clinical practices are key environments for skill acquisition during the education of nursing students, where it is important to encourage reflective learning. This study sought to explore the experience of final year nursing students during their clinical placement in emergency and intensive care units and to identify whether differences exist between female and male students. Using qualitative methodology, a documentary analysis of 28 reflective learning journals was carried out at a public university in Northern Spain. Four themes were identified: “an intense emotional experience”, “the importance of attitudes over and above techniques”, “identifying with nurses who dominate their environment and are close to the patient in complex and dehumanized units” and “how to improve care in critically ill patients and how to support their families”. The female students displayed a more emotional and reflective experience, with a strong focus on patient care, whereas male students identified more with individual aspects of learning and the organization and quality of the units. Both male and female students experienced intense emotions, improved their learning in complex environments and acquired attitudes linked to the humanization of care. However, the experience of these clinical rotations was different between female and male students.

## 1. Introduction

During undergraduate nursing studies, the acquisition of competencies, in a broad sense, is essential. In addition to theoretical and practical learning and the development of nursing attitudes, it is important to establish transversal competencies, such as leadership, communication, or interpersonal skills, as well as competencies for adequate personal and professional development. To achieve these transversal competencies, it is important to encourage reflection [1].

Clinical practices are an essential element of learning for nursing students [2,3], as they enable the application of theoretical knowledge in a real environment, the training of technical skills through interaction with patients and health workers and the development of nursing attitudes [4]. In addition, this is an ideal opportunity for students to reflect on their learning. Emergency departments and intensive care units (ICU) are clinical environments that encourage competence development; however, they also pose a challenge for students and teachers. These units are very complex, with high pressure to care for serious patients, which can negatively influence the students’ experience [5]. Careful planning, with guidance and follow-up by an instructor, are central elements in their development [6].

## 2. Background

Significant learning is not possible without reflection [7]. The reflective analysis of lived experiences or problems faced during professional practice can serve as a stimulus for learning [8]. A reflective attitude can be even more useful than technical mastery when dealing with changing situations in professional practice. During nursing training, reflective learning can take place through a reflective learning journal (RLJ). The RLJ is a written document in which students carefully analyze their thoughts, actions or interactions with others over a period of time [3,7]. Several studies have documented the usefulness of RLJs in enhancing the learning experience during clinical practice [9,10,11], stimulating professional development [12] and even personal development in the process of becoming a nurse [13,14,15]. In this way, RLJ can be used as an additional tool for teachers to assess students’ acquisition of nursing competencies during clinical education but also to learn about students’ personal experiences, including their coping strategies, thoughts, emotions and feelings [4,7,13].

Addressing emotions during clinical practice is very important [3,10,13]. When students are asked to keep an RLJ, they are voicing emotions experienced during clinical placements that are usually relegated to the context of individual students [10]. In addition, writing and reflecting on their feelings and emotions can also be therapeutic for students, since it allows them to stop and externalize their experiences [9,16], which increases their confidence in their ability to face future difficulties [13,17] and increases their capacity to empathize with patients and their families [18,19].

In addition, RLJs provide a “snapshot” of the daily reality on the clinical level (e.g., characteristics of services, type of patients, quality of nursing care, etc.) and the teaching process (e.g., student–nurse interaction, clinical practice schedules, etc.). Knowing the day to day life of emergency department and ICU from the perspective of students can be an enriching way to identify areas of improvement in these two nursing dimensions: clinical practice and teaching activity.

There is some prior research on the experience of students during the first year of clinical practice training [2,3,17,19,20]; however, there are fewer approaches to the experience of clinical placements in senior year students and in complex care settings, even though these may be more representative of how students will cope with the impending start of their professional development. In addition, we were unable to find any papers that compared clinical practice experiences by gender. Only a few studies include the student’s gender in the verbatim, without establishing a comparative analysis [9]. This is relevant because previous research has documented differences between male and female nursing students in relation to professional values [21], personal values [22], career choice and post-graduation outcomes [23]. In addition, socially constructed traditional gender norms can determine expected behaviors and attitudes in both male and female students in the context of a traditionally female profession [24]. During clinical practice, and through the RLJ, it is possible to observe whether there are differences in the preference or rejection of some activities based on gender—for example, whether men feel more attracted to management and coordination aspects or whether women identify more with the humanization of care, empathy with patients, etc., both of which are situations that are dictated by gender roles.

The aim of our study was to explore the experience of final year nursing students during their clinical practices in emergency department and ICU and to examine how this experience is interpreted by both female and male students. 

## 3. Materials and Methods 

### 3.1. Design

A qualitative study using documentary analysis of RLJs written by nursing students in their senior year during their clinical practices.

### 3.2. Participants and Setting

In Spain, the Degree in Nursing is a four-year university degree with 240 credits (in accordance with the European Credit Transfer and Accumulation System, ECTS). During the final year, students at the University of Oviedo (Spain) take a specific course on clinical practices in the emergency department and ICU (12 ECTS credits). All students must perform at least two clinical rotations, one in the emergency department and another in ICU, to complete 230 h of training. In addition, students must submit a clinical case study and an RLJ of the subject, which accounts for 10% of the final grade. During the 2017/2018 academic year, 78 students studied this course at the University of Oviedo (Spain). Twenty-eight RLJs were selected from the students (15 from women and 13 from men) who obtained the highest grades in the January 2018 evaluation.

Clinical practices took place in the emergency department and ICU of six public hospitals. The center where the largest number of students performed clinical practices (*n* = 20) was a level 3 public university hospital (1000 beds), where around 300 patients are seen daily in the emergency department and which has 75 ICU boxes. The remaining hospitals that received students were level 2 (<500 beds).

### 3.3. Research Team

The research team consisted of five nurses (four women and one man) from the University of Oviedo. The principal investigator had two years’ experience in critical care. She was a doctor, associate professor in nursing and the head of the clinical practicum subject. Three of the nurses had professional experience in emergency department and ICU and teaching experience in the Degree of Nursing. One researcher was also the teacher of the “Research in Nursing” subject at the University of Oviedo. The students knew the researchers through their participation in other subjects during the nursing degree. 

### 3.4. Instruments

In this course, students are required to complete a compulsory portfolio on clinical practices. The portfolio consists of two sections. The first was a descriptive section with administrative and clinical data on the clinical practices, including data on the hospital and the practice unit, type of pathologies and nursing activities performed. The second was a reflective part (RLJ) on the contributions of the placements to their learning, on the level of satisfaction with the clinical practicum and suggestions for improvement. Specifically, students are strongly required to reflect on the following competencies achieved in three areas (knowledge, skills and attitude): (1) providing nursing care to critically ill patients; (2) correctly performing the most common techniques in emergency department and ICU; (3) respecting ethical values related to privacy, confidentiality and respect for patients; (4) meeting the information and communication needs of patients and families. This is delivered by email at the end of students’ clinical practice training in a text document of unlimited length. At the beginning of the course, there is a two-hour face-to-face information session at the university on how to perform the RLJ, emphasizing its reflective nature, which must be more than just a description of the activities performed. Students were asked to engage in a reflective exercise concerning their daily actions [8] and to only record in their RLJ those aspects that were most relevant. They were given instructions on how to record each reflection, including the actions taken, the context, their emotions and how they could improve. In addition, several examples were provided. They were also encouraged to reflect on how the same activity could be done differently depending on the unit and the nurse carrying it out—for example, communication with a sedated patient.

### 3.5. Data Analysis

According to the methodology proposed by other authors [25,26], a three-phase content analysis of the RLJs was carried out. In the first phase, the texts were prepared for analysis. Within each journal, the sections “Identification of contributions to learning”, “Description of the competencies acquired (knowledge, skills and attitudes)” and “Suggestions for improvement” were selected. In the second phase, the information was organized and the actual content analysis was carried out [27]. The meaning units identified in the reports were assigned codes. The codes were then grouped and gathered into subcategories and categories. Finally, the main themes that summarized the students’ experience were formulated. According to the format of the RLJ, a previous thematic category, “knowledge, skills and attitudes competencies”, was used as a starting point; however, the remaining topics emerged after the documentary analysis. In the third “reporting” phase, the results were presented. The complete analysis process was presented (codes, subcategories, categories and themes) as well as the description or storyline of the results.

The analyses were carried out without the use of software. The analyses were conducted independently by two researchers and, after pooling the analyses, they were triangulated with the participation of another researcher from the group. 

### 3.6. Ethical Considerations

All students provided informed consent to the use of their journal for research purposes. Each participant was assigned a code to maintain anonymity, which was identified using “W” for women and “M” for men. Participation in this study had no influence on the grade assigned in their evaluation since this investigation was initiated months after the students completed the course. Our study was exempt from ethics committee approval, although it was conducted in accordance with the ethical standards set out in the original Declaration of Helsinki and its subsequent amendments.

## 4. Results

### 4.1. Experience of Nursing Students in Emergency Department and Intensive Care Units

Overall, the student experience reflected in the RLJs was positive in terms of learning, although a high emotional burden related to attendance at these units was noted. Most students perceived a high degree of coordination in these units and the importance of nurse/medical collaboration. 

**Figure 1:**
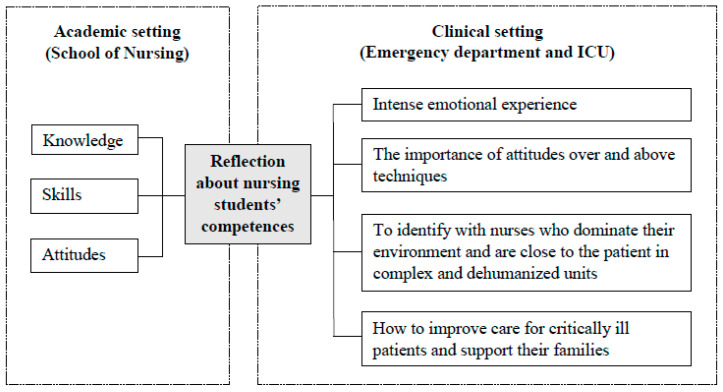
Experience of nursing students in emergency department and ICU during clinical practices.

During the analysis, four themes were identified from the students’ reflection on the competencies (Figure 1). The first referred to the student’s feelings of undergoing “an intense emotional experience” and the second referred to the skills and attitudes achieved “the importance of attitudes over and above techniques.” The third theme was related to nursing professionals and some characteristics of clinical practice units “to identify with nurses who dominate their environment and are close to the patient in complex and dehumanized units”. Finally, the fourth theme referred to patients and their families: “how to improve care for critically ill patients and support their families.”

#### 4.1.1. Intense Emotional Experience

For all the students, these clinical placements involved intense emotions. Prior to the clinical practices, negative emotions predominated, as the previous confrontation of female and male students was characterized by fear, pressure, emotional block, etc. However, during the clinical training, they experienced emotional ambivalence. Thus, they experienced positive feelings, especially linked to the patient’s favorable evolution and identification with the nursing profession. Concurrently, they also experienced negative emotions, associated with facing the care of patients in very serious clinical situations and death, causing them to reflect on life (Table 1).

As for the previous expectations, there were no great differences between girls and boys, in both groups, and feelings of fear, pressure, being “frozen” or blocked, etc. predominated. During the clinical practices, positive feelings of satisfaction and personal growth were expressed.

“These clinical practices have been a turning point in my career as I have been able to grow as a person and as a future nursing professional.”

W4

“In this clinical module I have shown myself how right I have been in choosing a profession like this, how close one is to the patient and how much chance one has of, with very little, improving the condition of the patient and his or her family.”

M10

Both the female and male students showed progressive confidence as the clinical practicum progressed, facing these with greater ease and feeling more satisfied if the patients progressed well.

Some acknowledged their difficulty in coping with death, either because of inexperience or because they felt overwhelmed by the situation. 

#### 4.1.2. Importance of Attitudes over and above Techniques

The acquired competencies were articulated in three areas: knowledge, skills and attitudes (Table 2).

In terms of theoretical knowledge, some categories common to both sexes were learning how to handle critically ill patients or how to prioritize emergency care through triage. They also recognized learning new and specific knowledge, required in these units. 

Regarding the competencies linked to skills or abilities, all mentioned the handling of devices and the refinement of new techniques as well as the improvement of other already known techniques. They also learned to act quickly, adjusting to the urgency of the moment. 

Attitude-related competencies were extensively analyzed as they constituted a very large section within the RLJs. Two themes were appreciated: firstly, in relation to their personal experience as students where the acquired responsibility or autonomy stands out; secondly, almost all referred to learning related to the humanization of care, based on respect for the patient, empathy and accompaniment.

“I have learned that many times there is no need to speak or, rather, “fill the silences” with words, we should simply be there, giving company and human touch if necessary.”

W12

“When the intubated patients were thirsty I would dip a gauze in water and place it between their lips and they would thank me. I also, for example, put the radio on for a patient because it’s quite tedious for everyone, I suppose, to be in bed all day without any entertainment.”

M12

In addition, students mentioned acquiring cultural competencies in dealing with people with social problems and patients from other countries/ethnicities.

“On the other hand, I have been in contact with people who are drug addicts as a result of a serious social problem and with a major underlying mental illness.”

W4

“Respecting the patients’ beliefs and cultures, always seeking their integration in the hospital.”

M7

#### 4.1.3. Identifying with Nurses Who Dominate Their Environment and Are Close to the Patient in Complex and Dehumanized Units

The perceptions of nursing professionals in the context of the emergency department and ICU are presented in Table 3. All the students appreciated the warm welcome to the units, the involvement of the nurses who taught them and their “willingness to teach”.

“I’ve discovered a part of nursing that’s exciting and that, if there’s one thing professionals have in this service, it’s passion and drive.”

W1

In addition, the students were grateful for the nurses’ attitudes.

“On a day-to-day basis in the special services, doubts and learning opportunities arose in which the nurses were always willing to help and explain things to me.”

M6

Both female and male students identified the emergency department and ICU as highly technical and labor-intensive environments. They also appreciated that nurses in these units worked more independently and autonomously than their colleagues in other units, e.g., inpatient units, and that they fulfill an important role in informing and reassuring patients:

“They are in control of the complexity of the situation at all times, always preventing it from overwhelming them.”

W3

“Nurses are not only the professionals who know how to inject, administer medication or put a bandage on. The most important thing is to know how to listen and be close to their patients, who at certain times only need someone close by, to feel their support and understanding.”

M7

#### 4.1.4. How to Improve Care in Critically Ill Patients and How to Support the Families

Both female and male students recognized that patients in these units presented very specific pathologies, which involved advanced practice care. They also understood the importance of informing and explaining the techniques to the patients in advance, as a measure to reassure them and avoid conflicts, especially due to long waits in the emergency department (Table 4). In this overall context, the students identified a certain depersonalization in patient care and proposed a more humane treatment, with simple verbal and non-verbal communication actions, such as calling each patient by their name or holding their hand. 

Regarding the families, all stressed the importance of adequately addressing their needs, creating a climate of trust and support, as they are under great pressure. The information that they receive plays an important role in this relationship as it can help to reassure them (Table 4). Both female and male students identified the importance of visiting times and how students and professionals should act during these encounters.

“Always showing them that we are there and that they can trust us to take care of their relatives.”

W3

“From my point of view I think it is important that the moment of family visits be as comfortable as possible for the relatives and the patient.”

M1

Lastly, several male and female students stressed the importance of reinforcing training on humanization in care before the start of the placements.

### 4.2. Differences in the Experiences of Clinical Practices in Critical Services by Gender

Female students reflected on a much more intense and negative emotional experience, linked to the environment of critical care practices with critically ill patients or by identification with young patients. Personal gratification after providing emotional care to patients was also common, as was concern for ensuring the best care:

“I have realized that if you treat them with affection and try to help them in any way you can, not only are they very grateful to you, but you also go home with a good feeling, and knowing that your work has served a purpose.”

W10

“Since we also usually have to make decisions or act very quickly which sometimes made me nervous because it can lead to confusion very easily.”

W13

However, for male students, perceived satisfaction was related to the identification of emergency department and ICU as an employment option.

“I must admit that it was the rotation that I enjoyed by far the most, especially in the area of emergencies, so I am seriously considering continuing to study to work in this type of care area in the future.”

M2

“The student’s autonomy has to take a step forward in order to prepare for the professional world.”

M8

In relation to competencies, the majority of female students stated that the techniques were not the most important aspect but rather the provision of basic care, such as those related to comfort, rest or pain relief.

“Anyone with training can channel a venous line or perform an electrocardiogram. As a nurse, you are there to support that person, reassure them, and accompany them in their distress. Sometimes the best cure is a smile, a hand on the shoulder or an “I’m there for you.””

W15

“Here, I learned how to take care of a patient, to keep an eye on him all the time, to wash him, to comb his hair, to take care of his nails... things that are less technical and more humane.”

W8

However, the male students gave importance to specific knowledge, such as how to change shifts properly or how to adapt to changing environments. The importance of teamwork was mentioned by virtually all male students.

“I see it as very important to be able to give shift changes in a proper manner. I have paid a lot of attention to those who, in my opinion, perform good shift changes and I have tried to assimilate this way of working.”

M3

“Teamwork, the willingness to always help one’s partner is one of the attitudes that I have encouraged during the rotation, there is no “so-and-so’s patient”, we are all there for everyone and we help each other with everything.”

M10

In terms of identifying with the nurses and the environment in these units, several female students reflected on attitudes that caused them to feel rejection and that they did not want to imitate.

“I have learned how I do not want to work in terms of how some health professionals describe and treat patients, not respecting their privacy, making value judgments and talking about patients in a derogatory way.”

W7

Finally, in relation to patients, the female students outlined the negative feelings perceived in patients with great detail (e.g., fear, nervousness, stress, worry) and the importance of ensuring intimacy.

“People who are conscious, in addition to their illness, are afraid and isolated and alone.”

W7

“From my point of view and according to what I have been able to learn while I was there, we can and must guarantee assistance, always respecting the patient’s physical and emotional intimacy.”

W3

## 5. Discussion

According to the results of our study, for the nursing students, clinical practice in the emergency department and ICU implied an intense emotional experience, demanding the importance of attitudes towards the techniques. The profile of the nurses highlighted their human nature and their role as a reference for students in these complex units with high care demands. They also identified the need to adequately inform and support critical patients and their families. Differences were detected between female and male students regarding the experience during this clinical training.

RLJs have been a useful tool for learning more about the experience of nursing students in complex units. Some authors [3,8] had already reflected on the usefulness of the journal as a method for venting and expressing feelings and for stimulating personal growth during the process of becoming a nurse [13,28]. Moreover, writing can be considered a therapeutic tool, in the same way as it is for patients admitted to ICU [29], and it can be useful for nursing students facing stressful situations such the emergency department and ICU [30,31]. 

The expectation surrounding these placements was very similar to that described by other authors: nervousness and worry before facing a new post, the need to apply knowledge and techniques previously explained in theory and progressive confidence and security over time [32]. In our case, this transition could be affected by the complexity and severity of the patients in specific services, which could accentuate the expression of negative feelings, which became evident during a very intense emotional experience [3,17,28].

The students expressed the same feelings that the patients displayed: nerves, fear, freezing up in urgent situations, etc. [32,33], as if they were acting as a mirror reflecting the same negative emotional reaction, whereas the nurses assumed a reassuring and controlling role in the situation, as identified by both male and female students. 

Student autonomy in complex services is limited [5], thus generating greater dependence on the nursing preceptor and greater observation of his/her clinical performance. The students in our study, as in other reports [34], have felt supported by the nurses during their clinical practices, who have integrated them into the team. During the process of becoming a nurse, which is more complex than simply having practical knowledge or skills [19,35], it is very important for students to have models and feel supported and accompanied during the mentoring process. The importance of this mentoring should be emphasized, not only through the nurse preceptors in the practice units but also on behalf of the university faculty, accompanying the students throughout the process, especially during the senior year [13]. To achieve this objective, it could be very useful to integrate this reflexive student learning into the creation of an environment of mutual trust and growth between teacher and student [10,12]. It is also important to consider the working conditions in these units, where high care loads and staff shortages can make it difficult for nurses to teach [36].

Both through the attitudes learned and the nursing care observed in the units, the humanization of care was essential. The students assigned great importance to respecting patients and the need to attend to their feelings and demonstrate empathy. Indeed, developing an empathic attitude is one of the fundamental pillars of the relationship between health professionals and patients/families [37]. Sensitivity to patient suffering was increased in our study since they were in a hostile environment, where patients were vulnerable, separated from their families and almost entirely dependent on the health professional, from making a rapid and effective diagnosis in the emergency unit to covering a human being’s basic needs in the ICU. Most of the plans for the ICU integrate the elements proposed by students, which is positive since it shows a growing awareness among future nursing professionals to improve the environment in these units [38].

In general, our results coincide with research conducted in Spain on RLJs as an assessment tool during the learning process of nursing students [11]. Many of the journals included reflections on the techniques but also frequently addressed patient and family related care, interactions with the team and the nurse preceptor and even death. Moreover, this study mentions very interesting aspects, such as the importance of providing clear guidelines for the implementation of these journals, the figure of the teacher as a guide in the process of reflection within the framework of a relationship of trust and the importance of assigning importance to the evaluation, in the understanding that, if it is proposed as a voluntary activity, students generally fail to participate.

In our study, a different experience was found among female and male students. The female students described their negative feelings in more detail during the clinical training and showed a more reflective attitude. This reality could be related to a greater ease of expressing emotions among the women, whereas the men may have had similar experiences or thoughts, although they may have ultimately decided not to include them in the journal or not to delve into the experience. 

Regarding the competences achieved, female students focused more on the humanization of care whereas male students more frequently mentioned aspects linked to their individual learning or organizational aspects in these units. This different approach by gender coincides with the results observed in nurses working in the ICU [39]. Further research is necessary to better understand these differences and to assess whether students may be imitating or reproducing the model identified by the nurse preceptors. 

Female students were also more concerned about poor patient progression or fear of making mistakes, which is consistent with the study by Fernandez-Feito et al., [21] where female students considered it more important to seek help when they could not meet patient needs.

### 5.1. Implications for Clinical Practice

Enhancing the use of these reflective tools can contribute to a better understanding of the experience of nursing students during their clinical placements and encourage personal and professional growth, which is difficult to achieve through theoretical training. In addition, it would be important to encourage male students to share their experience and their personal feelings. After being aware of this experience, it would be appropriate to design interventions to reduce the associated emotional impact and provide the students with strategies (e.g., coping styles) to reduce anxiety or negative feelings. 

In turn, teachers and nurse preceptors play a key role in accompanying the student in the process of “becoming” a nurse, and, in this process, creating and sharing a reflective narrative (student–preceptor–teacher) can be very helpful.

### 5.2. Limitations

In our study, the RLJ represented a percentage of the final grade. It would be very interesting to comment on the narratives with the students in a session not only aimed at modifying the assigned grade but also analyzing the overall experience in these services, as a group. The differences found according to gender should be interpreted with caution since the analysis was not performed blinded to the students’ genders, which may have biased the interpretation of the results. The results obtained were not subjected to the process of triangulation with other techniques, such as individual interviews or focus groups with students; this will be addressed in future research. 

## 6. Conclusions

For nursing students, clinical practices in emergency department and ICU represent an intense emotional experience that allows them to improve their learning and cope with complex environments. In addition to acquiring new knowledge and refining already learned techniques, these clinical practices allow students to acquire attitudes clearly linked to the humanization of care. Female and male students experienced these clinical rotations from different viewpoints. Female students were more emotionally and reflectively focused on patient care, whereas male students identified more with individual aspects of learning and the organization and quality of the units.

## Figures and Tables

**Table 1:** Main categories, sub-categories and codes about the theme “intense emotional experience”.

Main Codes	Sub-Categories	Categories
Fear of the unknown	Previous expectations	Feelings
Pressure		
Nervousness		
Emotional blockage		
Identification of severe young patients	Intense experience	
Tough experience		
Fatigue		
Impotence of not being able to communicate		
Feeling lucky		
Insecurity in complex patients	Emotions during clinical practices	
Fear of making mistakes		
Helplessness lack of time		
Helplessness poor patient evolution		
Satisfaction for good patient progress		
Gratification humanizing care		
Progressive safety/self-monitoring		
Feeling like a nurse	Identification with the nursing profession	Thoughts
Professional and personal enrichment		
Awareness of the importance of the nursing profession		
Satisfaction with correct performance of techniques		
Gratification of professional collaboration		
Feeling overwhelmed	Difficulty coping with death	
Fine line life/death		
Inexperience in facing death		
Irreversible change in a second	Reflecting on life	
Valuing what matters		
Temporality of human life		

**Table 2:** Main categories, sub-categories and codes about the theme “importance of attitudes over and above techniques”.

Main Codes	Sub-Categories	Categories
Identify situations of risk	How to act	Knowledge
Prioritizing by triage		
New and specific knowledge	Expanding knowledge	
Distribution of work/tasks according to professional profile		
Integrating theoretical knowledge		
Getting to know other cultures		
Performing new techniques	Technical skills	Skills
Specific techniques		
Refinement of already known techniques		
Care linked to infection prevention		
Rapid action		
Performing under pressure		
Only one aspect of care	Emphasis on techniques	
Relative importance		
Observation		
Responsibility	Acquired attitudes	Attitudes
Autonomy		
Confidentiality		
Teamwork		
Accepting errors		
Adapting to a changing environment		
Keeping calm		
Humanizing care		
Empathy	Regaining human values	
Guaranteeing privacy		
Paying attention to patient’s emotions		
Importance of talking and listening		
Respect		

**Table 3:** Main categories, sub-categories and codes about the theme “identify with nurses who dominate their environment and are close to the patient in complex and dehumanized units”.

Main Codes	Sub-Categories	Categories
A warm welcome	Attitude of nurses towards students	Attitude of the nurses
Motivation for teaching		
Integration of students into the team		
Control of complex environment	Nursing activity work environment	Nurses’ Actions
Autonomy/initiative		
Not overwhelmed		
Acting calmly in emergencies		
High pressure to provide care		
Reassuring attitude	Attending to patients	
Informational role		
Close ties to the patient and family		
Identifying nurses who are humane	Professional Identification: I want to be	Nurses as models
Learning by imitation of good practice		
Identifying each professional’s style		
Contempt towards patients	Professional rejection: I don’t want to be	
Poor education		
Highly technical services	Complex and dehumanized services	Characteristics of services
High complexity		
High care pressure (emergencies)		
Depersonalized services		
Paying attention to pain	As it should be	
Listening more to patients		
Quieter and less noisy environment		
Improving communication		
Emotional support		
Improve use of the emergency service		
Limiting mobile phone use		

**Table 4:** Main categories, sub-categories and codes about the theme “how to improve care for critically ill patients and support their families”.

Main Codes	Sub-Categories	Categories
Highly complex patients	Patient characteristics	Negative patient experience
Severity		
Concerned	Negative emotions	
Nervous		
Informing appropriately	Professional care	Necessary actions with patients
Favorable environment		
Conveying reassurance		
Making the effort to listen		
Humanization	Humanized care	
Facilitate rest and comfort		
Guaranteeing privacy		
Offering trust and support	Support for families	Actions with families
Reassuring		
Providing information		
Stressful situation		
Interaction with professionals/students	Visit	
Facilitating a pleasant environment		
Strong emotional impact		
